# Seroprevalence of TORCH Infections among high-risk pregnant women, Datia, Madhya Pradesh

**DOI:** 10.6026/973206300220940

**Published:** 2026-02-28

**Authors:** Abhishek Mehta, Abha Gupta, Puneet Agrawal

**Affiliations:** 1 Department of Microbiology, Government Medical College, Datia, Madhya Pradesh, India; 2 Department of Pediatrics, Government Medical College, Datia, Madhya Pradesh, India

**Keywords:** TORCH, seroprevalence, immunochromatography, rapid diagnostic test, pregnancy, Datia, Madhya Pradesh

## Abstract

The lack of data on the seroprevalence of TORCH infections among high-risk pregnant women in Datia, Madhya Pradesh, which is crucial for better maternal and fetal health outcomes, is a concern. Over six months, 50 serum samples were tested for IgM antibodies using immunochromatographic rapid card tests. Data show that 28% of women were positive for at least one infection, with CMV being the most common (16%). No co-infections were found. Thus, we show the effectiveness of ICT/RCT for rapid screening and emphasize the importance of TORCH screening in high-risk pregnancies.

## Background:

The acronym TORCH represents a group of perinatal infections: Toxoplasma gondii (T), Rubella virus (R), Cytomegalovirus (C), and Herpes Simplex Virus (H) that can cross the placenta and cause severe fetal damage [1]. These infections are often asymptomatic or present with mild, non-specific symptoms in the mother. Still, they can lead to devastating consequences for the fetus, including congenital malformations, neurological deficits, hearing and visual impairments, and fetal demise [2]. The burden of TORCH infections is not uniformly distributed and is influenced by geographical, climatic, and socio-economic factors. In India, the seroprevalence rates for these infections vary widely from region to region [3]. North Madhya Pradesh, including the Datia district, is a region with a mix of rural and urban populations, where factors like hygiene, sanitation, and awareness about prenatal care can influence disease transmission [4]. While definitive diagnosis often relies on sophisticated techniques like ELISA or PCR, their cost, technical expertise, and time-consuming nature make them less accessible in district-level hospitals and primary health centers [5]. Immunochromatographic Rapid Card Tests (ICT/RCT) offer a viable alternative for initial screening. They are inexpensive, provide results within 15-20 minutes, require minimal training, and do not need sophisticated equipment [6]. There is a paucity of published data on the prevalence of TORCH infections from the Datia region. Therefore, it is of interest to bridge this gap by determining the seroprevalence of TORCH IgM antibodies in a cohort of high-risk pregnant women using ICT/RCT as the primary diagnostic modality.

## Methodology:

This laboratory record-based, cross-sectional study was conducted at the Department of Microbiology, District Hospital, Datia, Madhya Pradesh, from January 2024 to June 2024. The study included 50 high-risk pregnant women, defined as those with a history of Bad Obstetric History (BOH), including recurrent spontaneous abortions, intrauterine death, or a previous child with a congenital anomaly, fever with rash during the current pregnancy, or close contact with cats/other pets (for Toxoplasma risk). Laboratory records of all such women referred for TORCH screening during the study period were included. Under aseptic precautions, 3 ml of venous blood was collected from each participant. The blood was allowed to clot and then centrifuged at 3000 rpm for 5 minutes to separate the serum, which was used immediately for testing. Serum samples were tested for IgM antibodies against Toxoplasma gondii, Rubella virus, Cytomegalovirus (CMV), and Herpes Simplex Virus (HSV-1 & HSV-2) using commercially available, CE-marked Immunochromatographic Rapid Card Test kits from M/s. J. Mitra & Co., India, following the manufacturer's instructions. The tests were interpreted as positive if both a control line and a test line appeared, and negative if only the control line appeared. As this was a laboratory record-based study, a waiver for individual consent was obtained from the Institutional Ethics Committee, and patient confidentiality was maintained. Data were entered into Microsoft Excel and analyzed using descriptive statistics (percentages) to determine the seroprevalence of the TORCH infections.

## Results:

A total of 50 serum samples from high-risk pregnant women were analyzed. The overall seroprevalence for recent/active TORCH infection (IgM positivity) was found to be 28% (14 out of 50). The distribution of infections showed that Cytomegalovirus (CMV) was the most prevalent agent, accounting for 16% of cases. This was followed by Herpes Simplex Virus (HSV) at 8% and Rubella at 4%. No sample was positive for Toxoplasma gondii IgM antibodies. All positive cases were for a single infection; no co-infections were identified in this study group (Table 1).

## Discussion:

This study provides a snapshot of the seroprevalence of recent TORCH infections in a high-risk obstetric population in Datia, North Madhya Pradesh. The overall IgM positivity rate of 28% indicates a substantial burden of active/recent infections in this cohort, warranting clinical and public health attention. The complete absence of Toxoplasma IgM (0%) is a notable finding. This could be attributed to the small sample size, dietary habits, or a genuinely low prevalence of acute toxoplasmosis in the pregnant population of this region. This contrasts with studies from Teimouri *et al.* (2020) [7], other parts of India, where Toxoplasma seroprevalence is higher. The 4% prevalence of Rubella IgM is concerning due to the teratogenic potential of the virus. This highlights that rubella susceptibility persists despite the inclusion of the Rubella vaccine in the national immunization program (MR vaccine), underscoring the importance of vaccinating adolescent girls and non-pregnant women of reproductive age as suggested by Thayyil *et al.* (2016) [8].

Cytomegalovirus (CMV) emerged as the most common pathogen (16%), consistent with studies across India that report CMV as a leading viral cause of congenital infections. CMV is ubiquitous, and transmission can occur through close contact with young children, saliva, and other body fluids [9]. The 8% seroprevalence for HSV IgM indicates active herpes infection, which poses a risk for neonatal herpes during delivery. This finding necessitates careful obstetric management, including the consideration of Caesarean section in cases with active genital lesions at the time of delivery [10]. The use of ICT/RCT in this setting proved highly effective. It allowed for rapid turn-around time, enabling clinicians to receive same-day results and initiate counseling and management promptly. While ELISA is considered more quantitative and sensitive, ICT serves as an excellent point-of-care screening tool in resource-constrained settings like a district hospital [10]. The small sample size (n=50) is a major limitation, and the findings cannot be generalized to the entire population. As a laboratory-based study, detailed clinical correlation of the positive cases was not available. Furthermore, ICT tests, while specific, can have lower sensitivity compared to ELISA, and false negatives are a possibility. The absence of IgG testing limits the understanding of the overall immune status of the population.

## Conclusion:

This study highlights a significant seroprevalence (28%) of recent TORCH infections among high-risk pregnant women in Datia, with CMV being the most common. The Immunochromatographic Rapid Card Test proved to be an effective and reliable screening tool. Routine TORCH screening, health education on preventive measures, and larger community-based studies are recommended.

## Figures and Tables

**Table 1:** Seroprevalence of Specific TORCH IgM Antibodies (n=50)

**Pathogen**	**Number of Positive Samples**	**Seroprevalence (%)**
Toxoplasma gondii	0	0%
Rubella Virus	2	4%
Cytomegalovirus (CMV)	8	16%
Herpes Simplex Virus (HSV)	4	8%
Total Positives	14	28%
